# Iron‐inhibited autophagy via transcription factor ZFP27 in Parkinson's disease

**DOI:** 10.1111/jcmm.17946

**Published:** 2023-09-05

**Authors:** Yinying Wang, Qian Wen, Rongsha Chen, Zhichao Gan, Xinwei Huang, Pengfei Wang, Xia Cao, Ninghui Zhao, Zhongshan Yang, Jinyuan Yan

**Affiliations:** ^1^ Center Laboratory of the Second Hospital affiliated Kunming Medical University Kunming China; ^2^ Neurosurgery Department of the Second Hospital Affiliated Kunming Medical University Kunming China; ^3^ Yunnan Provincial Key Laboratory of Molecular Biology for Sino Medicine Yunnan University of Chinese Medicine Kunming China

**Keywords:** autophagy, IGF2, iron, Parkinson's disease, ZFP27

## Abstract

Parkinson's disease (PD) is a challenge because of the ageing of the population and the disease's complicated pathogenesis. Accumulating evidence showed that iron and autophagy were involved in PD. Nevertheless, the molecular mechanism and role of iron and autophagy in PD are not yet elucidated. In the present study, it was shown that PD mice had significant motor dysfunction, increased iron content, less dopamine neurons and more α‐synuclein accumulation in the substantia nigra. Meanwhile, PD mice treated with deferoxamine exhibited less iron content, relieved the dyskinesia and had a significant increase in dopamine neurons and a significant decrease in α‐synuclein. Autophagy induced by LC3 was inhibited in PD models with iron treatment. Following verification showed that iron aggregation restrained insulin‐like growth factor 2 (IGF2) and transcription factor zinc finger protein 27 (ZFP27) in PD models. In addition, LC3‐induced autophagy flux was reduced with ZFP27 knockdown. Furthermore, ZFP27 affected autophagy by regulating LC3 promoter activity. These data suggest that iron deposition inhibits IGF2 and ZFP27 to reduce LC3‐induced autophagy, and ultimately decrease dopamine neurons, accelerating PD progression. Our findings provide a novel insight that ZFP27‐mediated iron‐related autophagy and IGF2 may activate the downstream kinase gene to trigger autophagy in the PD model.

## 1 INTRODUCTION

With the ageing of the population, the incidence and prevalence of neurodegenerative diseases are rising yearly, Parkinson's disease (PD) is the second most common neurodegenerative disease; its clinical manifestations include myotonia, bradykinesia, static tremor and other symptoms.^1^ PD can be caused by genetic factors, environmental factors, ageing, cerebrovascular diseases, poisoning, infections and other factors.^1^ The primary pathological features of PD are selective death of dopaminergic neurons in the substantia nigra (SN) and accumulation of α‐synuclein (α‐syn). Studies showed that PD is associated with oxidative stress, abnormal mitochondrial function, lack of ubiquitination protease degradation system, neuronal apoptosis, synaptic transmission disorders and intracellular calcium homeostasis imbalance.^2^ The clinical manifestations, pathological changes and pathogenesis of PD‐related genes have been studied, and treatments include medications, surgery, stem cell therapy and gene therapy; these treatments only temporarily improve symptoms and do not prevent or delay disease progression.^1^, ^3^ The main reason is that the mechanism of PD is still not entirely clear. Hence, further study on the molecular mechanism of PD is critical. A growing literature about neurodegenerative diseases indicated that imbalances of metal ion homeostasis exacerbate oxidative stress, and lead to neuronal death.^4^, ^5^ Iron is an essential trace metal element that mediates several biological functions of the body.^6^ Iron is not only involved in neural tissue metabolism, energy supply, DNA replication and cell cycle, but also involved in myelin synthesis, metabolism and neurotransmitter synthesis.^7^ Nevertheless, excessive iron accumulation leads to plasma ceruloplasmin deficiency and neuroferritin lesions, which are neurodegenerative changes caused by excessive deposition of iron.^8^, ^9^ Iron injection into the brain of animals also induced behavioural changes and symptoms of PD.^10^, ^11^ The iron accumulation in the SN via the deletion of the Iron regulatory protein 2 exacerbated dopaminergic neuron apoptosis and Parkinsonism symptoms.^12^ High dietary iron increased the death of dopaminergic neuron, and reduced the content of dopamine in transgenic mice with overexpressing human A53T α‐syn.^13^ A large accumulation of iron can produce reactive oxygen species, increase oxidative stress and cause mitochondrial dysfunction and misfolding of α‐syn; of these, oxidative stress is considered to be one of the primary causes of iron overload leading to PD.^14^, ^15^, ^16^, ^17^, ^18^, ^19^ However, antioxidants do not cure PD, suggesting that iron overload may involve other mechanisms that accelerate the onset and progression of PD. These findings suggest that the molecular mechanism of iron‐mediated PD needs further elucidation.

Autophagy is highly conserved in eukaryotes. As a scavenger in cells, autophagy wraps macromolecules, long‐lived proteins and damaged organelles into autophagosomes degraded by lysosomes, to maintain cellular homeostasis.^20^ Studies showed that autophagy plays a crucial role in neuron survival and neurodegenerative degeneration.^21^ Dysfunction of autophagy causes the aggregation of misfolded proteins and damaged organelles, eventually leading to neurodegenerative diseases such as Huntington's disease, amyotrophic lateral sclerosis (ALS), Alzheimer's disease (AD) and PD.^21^ Studies found that autosomal gene mutations in patients with inherited PD were related to autophagy‐related genes.^22^ Autophagy occurred significantly less in PD than in normal controls, and Beclin1 was also reduced in PD cell models overexpressing α‐syn.^23^, ^24^ Microtubule‐associated protein 1 light chain 3 (LC3)‐II expression was dramatically lower accompanied by mitochondrial dysfunction in a PD mice model with DJ‐1 mutations.^25^ These findings suggest that enhancing autophagy would be a treatment strategy for PD. 1‐Methyl‐4‐phenyl‐1,2,3,6‐tetrahydropyridine (MPTP)‐induced PD mouse models treated with the autophagy activator rapamycin markedly increased dopamine neurons and decreased α‐syn aggregation.^26^ Similarly, activation of autophagy via Beclin1 overexpression alleviated PD signs in mice.^24^ Corynoxine B derivative CB6 improved autophagy against PD by activating the PIK3C3/VPS34 complex.^27^ Improved autophagy by neuropeptide Apelin‐13 also alleviated the behavioural dysfunction and increased dopamine neurons.^28^ All the above studies suggest that autophagy is involved in PD.

Excessive iron can aggravate PD symptoms by increasing oxidative pressure, abnormal mitochondrial function and α‐syn aggregation. In addition, autophagy participates in PD. Iron overload increased α‐syn accumulation by autophagy inhibition in both cultured neurons and SH‐SY5Y cells.^29^ The iron chelation agent deferoxamine (DFO) induced autophagy to prevent neuronal injury in the PD cell model.^30^, ^31^ Conversely, it is reported that chronic iron could increase autophagy to promote cell death in cells expressing the A53T α‐syn.^32^ To date, the relationship between iron and autophagy in PD remains unclear. Therefore, we performed this study to determine whether and how iron modulates autophagy in PD.

Here, we focused on the interaction mechanism of iron and autophagy in PD using mouse and cell model. We found that iron deposition suppressed autophagy to decrease TH‐positive neurons and cell viability. Thereafter, iron attenuated the transcription factor a zinc finger protein 27 (ZFP27) and insulin‐like growth factor 2 (IGF2) to reduce autophagy and ultimately lead to PD aggravation.

## 2 MATERIALS AND METHODS

### 2.1 Animals and drug treatments

**FIGURE 1:**
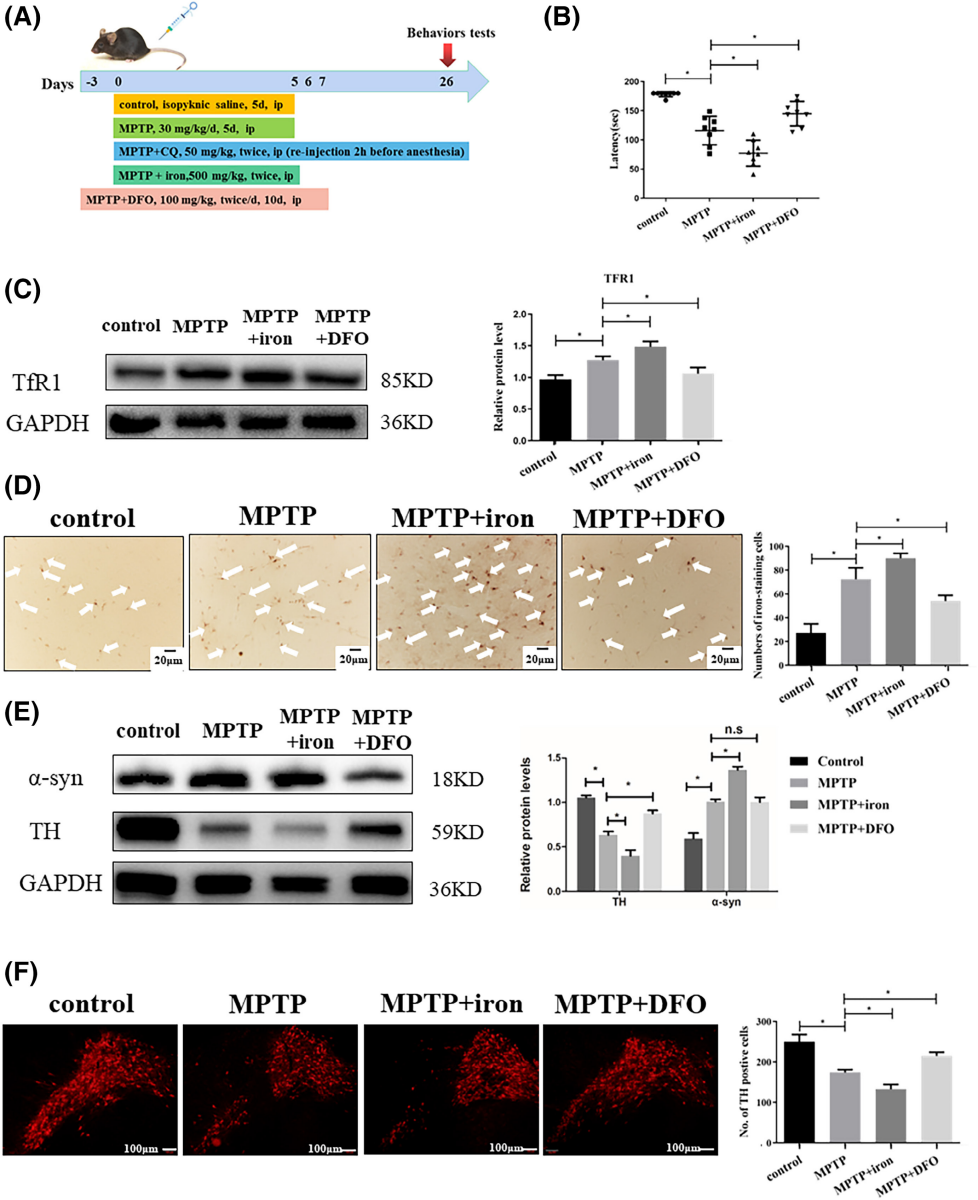
Excessive accumulation of iron accelerates the progression of Parkinson's disease. (A) The schematic diagram of the timeline about control, MPTP‐induced PD model, PD model with CQ treatment, PD model with iron treatment, PD model with DFO treatment. (B) Excess iron aggravated dyskinesia in PD mice according to the rotarod test. (C) Transferrin receptor (TfR1) levels were higher in PD mice than in the control mice (western blot; GAPDH served as the internal control); The relative grey intensity of TfR1 was analysed using Image J. (D) The change of iron levels in the brains of all groups mice (Perl's staining; scale bar = 20 μm). (E) Iron deposition markedly reduced the expression of tyrosine hydroxylase (TH) and increased α‐syn accumulation in the SN; The relative grey intensity of TH and α‐synuclein were analysed using Image J. (F) Iron significantly lessened the number of TH‐positive dopaminergic neurons, and DFO rescued the expression of TH in the SN (immunofluorescence staining; scale bar = 100 μm). All data are presented as the mean ± SEM, ns *p* ≥ 0.05; *p < 0.05.

Male C57BL/6J mice (weight 18–25 g, 8 weeks old) were obtained from the Animal Center, Kunming Medical University, China. All mice were fed in a controlled environment and provided standard rodent chow and water. The implementation of animal care and procedures was approved. All mice were randomly divided into the following five groups: control group, MPTP (MPTP‐HCl, Sigma, M0896, 30 mg/kg) group, MPTP+ chloroquine (CQ, Sigma, C6628, 50 mg/kg) group, MPTP+ iron (iron dextran, Sigma, D8517, 500 mg/kg/day) group, MPTP+DFO (Sigma, D9533, 100 mg/kg) group (Figure 1A). The mice of the control group were administered with intraperitoneal (ip) injection saline for five consecutive days. For the MPTP group, the mice were ip injected with MPTP‐HCl for five consecutive days. For the MPTP+CQ group, the mice were administered via ip injection with MPTP‐HCl for five consecutive days, then ip injection with CQ and finally re‐injection of CQ 2 h before euthanasia. The mice of the MPTP+ iron group were treated by ip injection of MPTP‐HCl for five consecutive days, and then ip injection of iron dextran once daily for 2 days. The dose of iron dextran is based on the references.^33^, ^34^ In the MPTP+ DFO group (iron deprivation group), the mice were also treated with ip injection of MPTP‐HCl for 5 days, and ip injection of DFO twice daily for 10 days (from 3 days before until up to 7 days after the start of MPTP) according to the literature.^35^ All the mice were euthanized 21 days after the last MPTP injection.

### 2.2 Western blot

After the mice were euthanized, these SNs were dissected for determining the protein and mRNA levels. The SN of the mice and SH‐SY5Y cells with or without treatment were harvested and lysed in RIPA lysis containing protease inhibitor cOmplete Tablets, Mini EDTA‐free, EASY pack (Roche, 04693159001). The protein samples were subjected to 12.5% SDS‐PAGE gel electrophoresis and transferred to the PVDF membrane in a BIO‐RAD transfer apparatus, then blocked in 5% skimmed milk for 1 h. The membrane was incubated overnight at 4°C with the primary antibody, including GAPDH (Proteintech, 10494‐1‐AP, 1:10,000), TH (Proteintech, 25859‐1‐AP, 1:5000), α‐syn (Cell Signalling Technology, D37A6, 1:1000), LC3 (Sigma, L7543, 1:1000), p62 (Sigma, P0067, 1:1000), ZFP27 (Antibodies, 2780268, 1:500), IGF2 (Immunoway, YN1761, 1:500), TfR1(abcam, ab214039, 1:1000), Gm4724 (biorbyt, A81254, 1:500). Then, the membrane was followed by three times of wash with TBST and incubated with target secondary antibody—KPL Peroxidase‐Labelled Affinity Purified Antibody To Rabbit IgG (H + L) (KPL Affinity Purified Antibody, SeraCare, 1:10,000) for 2 h at room temperature. After three times washing, bands were visualized by using the enhanced chemiluminescence star (ECL, Beyotime, P0018FS). The housekeeping GAPDH protein as an internal standard was used for normalization. The protein band intensity was quantified using Image J Software 9.

### 2.3 QPCR

Total RNA was extracted from SN of mice using TRIzol reagent (Invitrogen) following the instructions of the manufacturer. A NanoDrop2000 Spectrophotometer (Thermo Fisher Scientific) was used to quantify the RNA concentration. For assessments of mRNA expression, 1 μg total RNA was used to synthesize the complementary DNA using a PrimeScript™ RT reagent kit with gDNA Erase (Takara, RR047B), QPCR was performed using TB Green Premix Ex Taq™ II (Tli RNaseH Plus) (Takara, RR820A). The sequences of primers are presented in Table 1. The relative mRNA levels were calculated in accordance with 2^−ΔΔCt^ values using GAPDH as the internal control gene.

**TABLE 1:** The PCR primers used in this study.

Gene	Forward primer (5′–3′)	Reverse primer (5′–3′)
ZFP27	GAGGATGAGCCGAAGCATAGT	GGTCCGGTAATTTCTCTCCTGT
Gm4724	GTTCTGCAGAGCAACCCTCT	TGCAGGACATTCCATGTTCTAAAG
IGF2	CGTGGCATCGTGGAAGAGT	ACGTCCCTCTCGGACTTGG
Fgfrlop	CCCATCGCTAACAGATCCAGA	GTTCCCGTCCTCTACTCCCA
WDCP	GAGTCACTCCCTATCCTTCCTC	GGGTCCAACAAGCACAGTAAAC
Myo5A	GAAGTGTGGAAATCGGCAGAG	ATGTCAGGGTTCCGTAAGTGA

### 2.4 Immunofluorescence (IF) staining

All anaesthetised mice were perfused through 4% paraformaldehyde. Perfused brain tissue was sliced into coronal semi‐serial 30 μm thick sections of the SN using a cryostat (Thermo), and brain slices containing the major portion of the SN was chosen to be stained with the primary antibody LC3 or α‐syn overnight at 4°C. The sections were then incubated for 2 h with the secondary antibody Alexa Fluor 488 goat anti‐rabbit lgG (H + L) or directly labelled antibodies Alexa Fluor594 anti‐TH Antibody. Finally, DAPI (abcam, ab104139) staining solution was added to the samples. The images were acquired using a fluorescence microscope (BX51, Olympus) equipped with a digital camera (DP73, Olympus) or a laser scanning confocal microscope (FV3000, Olympus Biosystems). The antibodies were used in our experiment were as follows: TH (1:500, Biolegend, 818003), LC3 (1:1000, Sigma, L7543), Alexa Fluor 488 goat anti‐rabbit lgG (H + L) (1:1000, Invitrogen, A11034), α‐syn (abcam, ab138501, 1:600), LAMP1 (ABclonal, A2582, 1:100).

### 2.5 Rotarod test

The rotarod test was conducted to evaluate rodent motor coordination. The method is modified from a previous study,^36^, ^37^ all mice were trained in advance on the rotarod apparatus in order to reach a stable performance before the first injection of MPTP. The training consisted of four sessions on 3 consecutive days, under an accelerating protocol speed from 0 to 30 rpm in 180 s; each session included three separate trials, with at least 5 min of rest between trials. The length of time that the mice managed to remain on the rod was recorded 21 days after the last MPTP injection. The average of the three trials was used for further analysis.

### 2.6 Perls' staining

The cryosections were incubated in 2% potassium ferrocyanide with 2% HCl (1:1) for 30 min, washed with PBS for 5 min three times and subsequently treated with 1% H_2_O_2_ in methanol for 20 min. After washing with PBS three times, the slides were incubated with diaminobenzidine (DAB). Finally, washed with distilled water, the sections were mounted on microscope slides, dried, coverslipped and observed by microscope.

### 2.7 Transmission electron microscopy (TEM)

Under anaesthesia, the fresh SN of mice were harvested and fixed with 2.5% glutaraldehyde, post‐fixed with 1% OsO4 in 1% PB (pH 7.4), dehydrated in a graded series of ethanol, and embedded in resin, cut to 60–80 nm ultrathin section and finally stained by standard procedures. Autophagosomes were observed under a HT7800 transmission electron microscope (Hitachi).

### 2.8 Cell culture and drug treatments

SH‐SY5Y and HEK293T cell lines used in this study were obtained from Kunming Institute of Zoology. The SH‐SY5Y cells were maintained in DMEM (Gibco) cell culture medium supplemented with 10% foetal bovine serum (Gibco), 1% Penicillin/Streptomycin (Gibco) and standard cell culture condition (37°C, 5% CO_2_) was employed at all time during the study. The medium was replaced every day until the cells reached 80%–90% confluency. The HEK293T cell line is widely used to explore the functions of target genes after transfection assays. HEK293T cell was cultured in the DMEM medium (Gibco) with 10% foetal bovine serum (Gibco) and 1% (v/v) penicillin–streptomycin (Gibco) at 37°C in humid atmosphere with 5% CO_2_. The medium was changed every day in all experiments.

The SH‐SY5Y cells are treated with MPP^+^ to induce vitro PD model. Then, the SH‐SY5Y cells were divided into five groups: control group (no treatment group), 1‐methyl‐4‐phenylpyridinium (MPP^+^, Sigma, D048) group (PD model group), MPP^+^+CQ group, MPP^+^+iron group and MPP^+^+DFO group. For the MPP^+^ group, the cells were treated with 1 mM MPP^+^ for 24 h. In MPP^+^+CQ, MPP^+^+iron or MPP^+^+DFO group, the cells were separately treated with 10 μM CQ, 100 μg/mL FAC or 100 μM DFO for 12 h before 1 mM MPP^+^ treatment for 24 h.

### 2.9 Cell transfection assays

For knock‐down experiments, overexpression experiment and luciferase assay, HEK293T cells were transfected with the pLKO‐1‐Puro plasmid as negative control (shRNA‐NC), shRNA‐ZFP27 with the pLKO‐1‐Puro plasmid, pLVX‐IRES‐mCherry empty plasmid as negative control (NC), IGF‐2 overexpression (IGF‐2 OE) with the pLVX‐IRES‐mCherry‐IGF2, some reporter plasmids (pGL3‐basic or pGL3‐LC3) and so on. The 4 μg plasmids were mixed and incubated with 10 μL Lipofectamine 2000 (Invitrogen, 11668019) in DMEM medium for 20 min at room temperature. The DNA‐Lipofectamine 2000 complexes were added to a 6‐well culture plate with HEK293T cells, and cultured for 48 h with or without treatments. Then, the cells were collected and processed for further detection according to the experimental design.

### 2.10 Luciferase assay

The HEK293T cells were seeded on a 6‐well culture plate and transfected with a normalized vector (pRL‐TK), a reporter vector (pGL3‐basic or pGL3‐LC3), shRNA‐ZFP27 and the corresponding negative control (shRNA‐NC). The cells were divided into the following groups: (1) pRL‐TK and pGL3‐basic; (2) pRL‐TK, pGL3‐LC3 and shRNA‐NC; (3) pRL‐TK, pGL3‐LC3 and shRNA‐ZFP27; (4) pRL‐TK, pGL3‐LC3 and shRNA‐NC with 100 μg/mL FAC for 12 h; (5) pRL‐TK, pGL3‐LC3 and shRNA‐NC with 100 μM DFO for 12 h. After transfection for 48 h, the cells were plated in 96‐well plates at no less than 2 × 10^4^ cells per 75 μL for Dual‐luciferase reporter gene assay. The luciferase activities were measured via Dual‐luciferase assay system (Promega, E2920) according to the manufacturer's instructions using an in vivo bioluminescence imaging system.^38^ All assays were replicated for at least three times.

### 2.11 Autophagy flux

The HEK293T cells were seeded on a 6‐well culture plate with cell crawling pieces and transfected with shRNA‐NC, shRNA‐ZFP27 or mRFP‐eGFP‐LC3 plasmid to detect autophagy flux. After transfection for 48 h, the cells were taken out and stained with DAPI (abcam, ab104139). Images acquisition of the green and red puncta was collected by a laser scanning confocal microscope (FV3000, Olympus Biosystems).

### 2.12 Transcriptome sequencing analysis

The mice SN from the PD group and PD with the iron group were collected, and then total RNA was extracted using TRIzol (Invitrogen) according to the manufacturer's instructions. We employed the Biomarker Technology Company to perform RNA sequencing on the Illumina platform. Differentially expressed genes were obtained using a threshold a log2 fold change ≥1.5 and *p* < 0.05 between MPTP and MPTP+iron groups.

### 2.13 Statistical analysis

All of the data are expressed as the mean ± SEM. Statistical significance was determined using GraphPad Prism 9. Data were analysed from two experimental groups using a two‐tailed Student's *t*‐test, and analysed from three or more groups using a one‐way analysis of variance (anova) followed by Dunnett's multiple comparisons test. At least three independent trials in this paper were performed. no significant (ns) *p* > 0.05; *p < 0.05.

## 3 RESULTS

### 3.1 Iron exacerbated PD

In neurodegenerative diseases, dyshomeostasis of metal ions exacerbates oxidative stress and leads to neuronal death. Clinical studies showed that iron levels are significantly higher in the brains of PD patients than in healthy people, and iron in SN neurons and glial cells positively correlated with the severity of PD.^39^, ^40^ Therefore, we evaluated the effects of iron in the PD mice model produced by MPTP treatment. In the rotarod test, the fall latency of the MPTP group was lower than in the control group (Figure 1B). We also observed a significantly lower latency in PD mice treated with iron compared to the MPTP group, and the fall latency was enhanced in DFO‐treated PD mice (Figure 1B). We then determined whether the change in motor function was due to the iron. The transferrin receptor 1 (TfR1) maintains iron homeostasis into the cell, and the expression of TfR1 is closely correlated with iron content.^41^, ^42^ As shown in Figure 1C, TfR1 expression was higher in the MPTP group than in the control group, and the TfR1 expression was lower after DFO treatment with PD mice. The iron content by Perls' staining was significantly higher in MPTP‐induced model than in controls. In treatment with DFO in PD mice, the iron of SN exhibited a reduced trend (Figure 1D). Tyrosine hydroxylase (TH) is the rate‐limiting enzyme in dopamine synthesis and the specific marker of dopaminergic neurons. We observed that TH protein levels were obviously lower in the PD mice with iron relative to that of MPTP‐induced PD mice (Figure 1E). As the reducing iron by DFO, TH protein levels showed a partial recovery (Figure 1E). An obvious increase in the accumulation of α‐syn in iron treated with PD mice was observed relative to that of PD model animals; however, the expression of α‐syn in the DFO treated with PD group was not significant (Figure 1E). We also observed a lower number of TH‐positive cells in SN for animals administered with iron compared to no iron group (PD group) by immunofluorescence staining (Figure 1F). More TH‐positive neurons were observed with DFO than the PD group with no DFO (Figure 1F). Taken together, these data suggest that iron treatment has a detrimental effect on PD mice.

### 3.2 Iron‐induced autophagy aggravated PD

Excess iron causes stress on physiological functions in cells. Autophagy is critically involved in stress responses. Autophagy participates in maintaining iron homeostasis.^43^, ^44^ Iron chelators mediate autophagy signalling in PD.^30^ Then, we explored the relationship between iron and autophagy in PD for these reasons.

**FIGURE 2:**
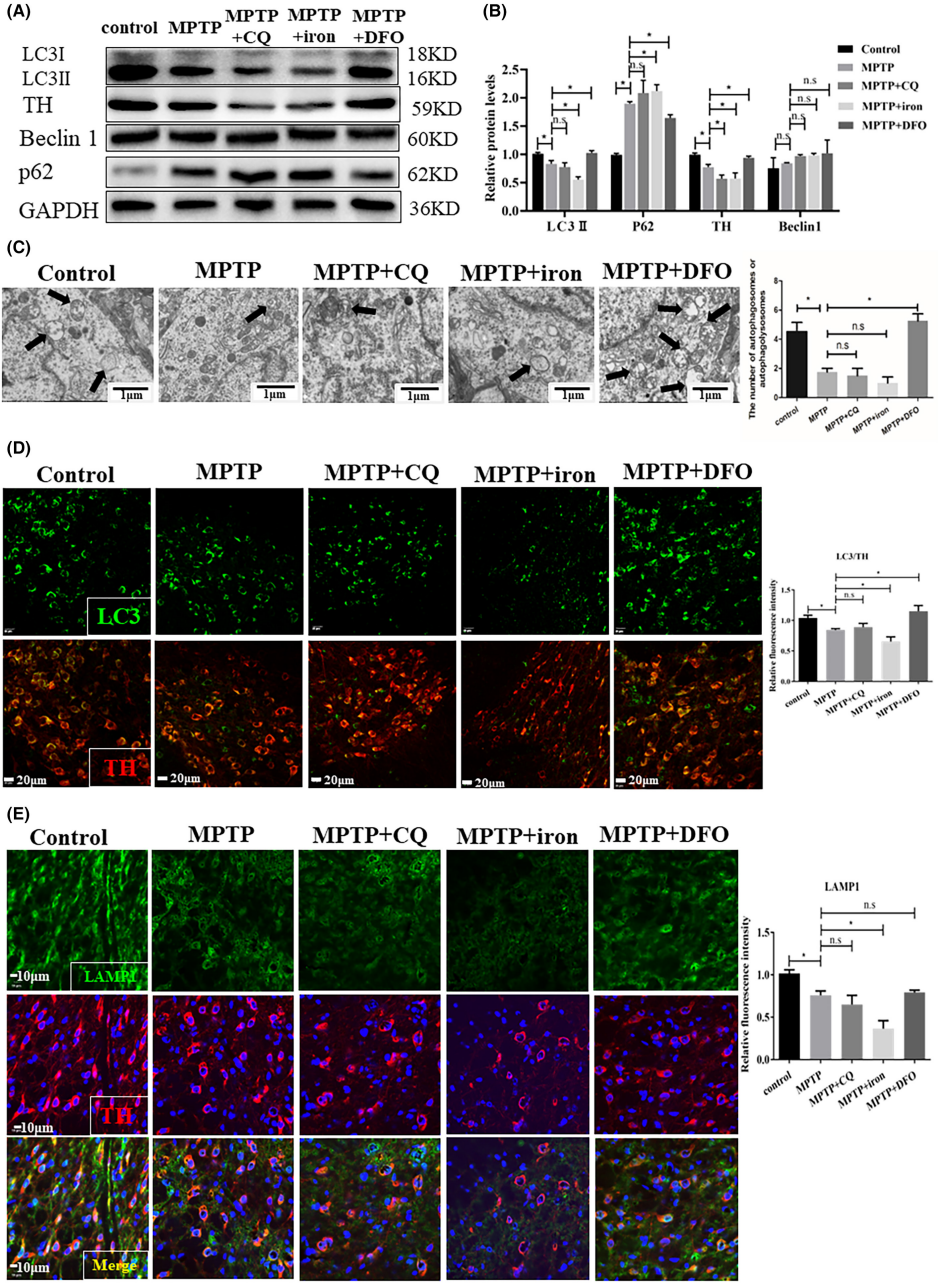
The change of autophagy via iron deposition and iron deprivation in SN of PD mice. (A, B) Western blot results and quantification analysis of autophagy‐related genes LC3, p62, Beclin1 protein expression in control, MPTP, MPTP+CQ, MPTP+iron and MPTP+DFO groups (relative to the internal control GAPDH). (C) Representative transmission electron microscopy images represented autophagosomes marked with the black arrow in the SN of all groups (magnification ×8000; scale bar, 1.0 μm); The numbers of autophagosomes or autophagolysomes were analysed using Image J software. (D) Double immunofluorescence staining of TH (red) and LC3 (green) in the SN of five groups mice (scale bar: 20 μm); relative fluorescence intensity was analysed using Image J. (E) Double immunofluorescence staining of TH (red) and LAMP1 (green) in the SN of all groups mice (scale bar: 10 μm); relative fluorescence intensity was analysed using Image J. All data are presented as the mean ± SEM, ns *p* ≥ 0.05; *p < 0.05.

Firstly, we measured an autophagy marker LC3II to monitor autophagy. When autophagy was activated, more cytoplasm LC3I was converted to LC3II on the membrane of the autophagosomes. In the MPTP‐induced PD mice, we found that LC3II but not Beclin 1 was obviously decreased in the SN (Figure 2A,B). To assess whether iron is responsible for autophagy, we investigated the role of iron in PD mice. As shown in Figure 2A, MPTP‐induced PD mice with the addition of iron decreased LC3II expression and increased an autophagic substrate p62 expression, but DFO treatment significantly activated autophagy than PD mice without treatment (Figure 2A,B). CQ is an autophagy inhibitor by blocking autophagosome‐lysosome fusion and increasing LC3II expression level. The LC3II in PD models received CQ was obviously reduced relative to the CQ alone group (Figure S1), indicating that autophagy flux was restrained in MPTP‐induced mice. Besides, LC3II expression of CQ treatment mice in SN had an increasing trend but no difference relative to the control mice, but CQ treatment could improve LC3II levels in the cell model (Figure 2A,B; Figure S1). And, TH protein levels of CQ mice were decreased compared to the control (Figure S1B), the result approved that CQ might penetrate into the brain to mediate the survival rate of dopamine neurons. It was suspected that the change of LC3II in mice neurons may be attenuated due to the SN tissue containing neurons, glia cells and nerve fibre, and so on. MPTP/MPP^+^‐induced PD model caused severe autophagy damage, and the inhibition of MPTP/MPP^+^ on LC3II was stronger than the enhancement of CQ on LC3II (Figure S1A,C). Combined with the data of TH protein level, the effect of MPTP was also stronger than CQ (Figure S1B). Hence, LC3II expression of the MPTP+CQ group showed no significant difference with the MPTP group, the result was consistent with cell model (Figure 2A,B; Figure S1). Moreover, we also found that TH protein levels in MPTP+CQ mice were obviously lower than in the MPTP‐alone group. This demonstrated that both CQ and MPTP could inhibit autophagy for increasing the death of TH‐positive neurons in SN (Figure 2A,B; Figure S1). In addition, double membrane‐bound autophagosomes or autophagolysosomes (marked with black arrows) were lower after treatment with MPTP than in the control mice by transmission electron microscopy (TEM) in Figure 2C. On the contrary, the MPTP+DFO group raised the number of autophagosomes and autophagolysosomes (Figure 2C). Double immunofluorescence staining (LC3/TH) also showed fewer accumulated LC3‐positive granules, and fewer TH‐positive cells in the MPTP+iron group (Figure 2D). The autophagosomes in dopamine neurons were rescued with DFO treatment than in the PD model without treatment (Figure 2D). Lysosomal‐associated membrane protein1 (LAMP‐1) is a lysosomal outer membrane protein and is used as a lysosome marker to monitor autophagy flux. We found that the PD mice with iron decreased the expression of LAMP1 compared with PD mice by IF staining (Figure 2E). These findings suggest that iron suppressed autophagy flux by LC3II but not Beclin 1 in the PD model and that reduced autophagy is involved in the PD occurrence and progression.

### 3.3 ZFP27 and IGF2 were involved in iron inhibition of autophagy in PD model

**FIGURE 3:**
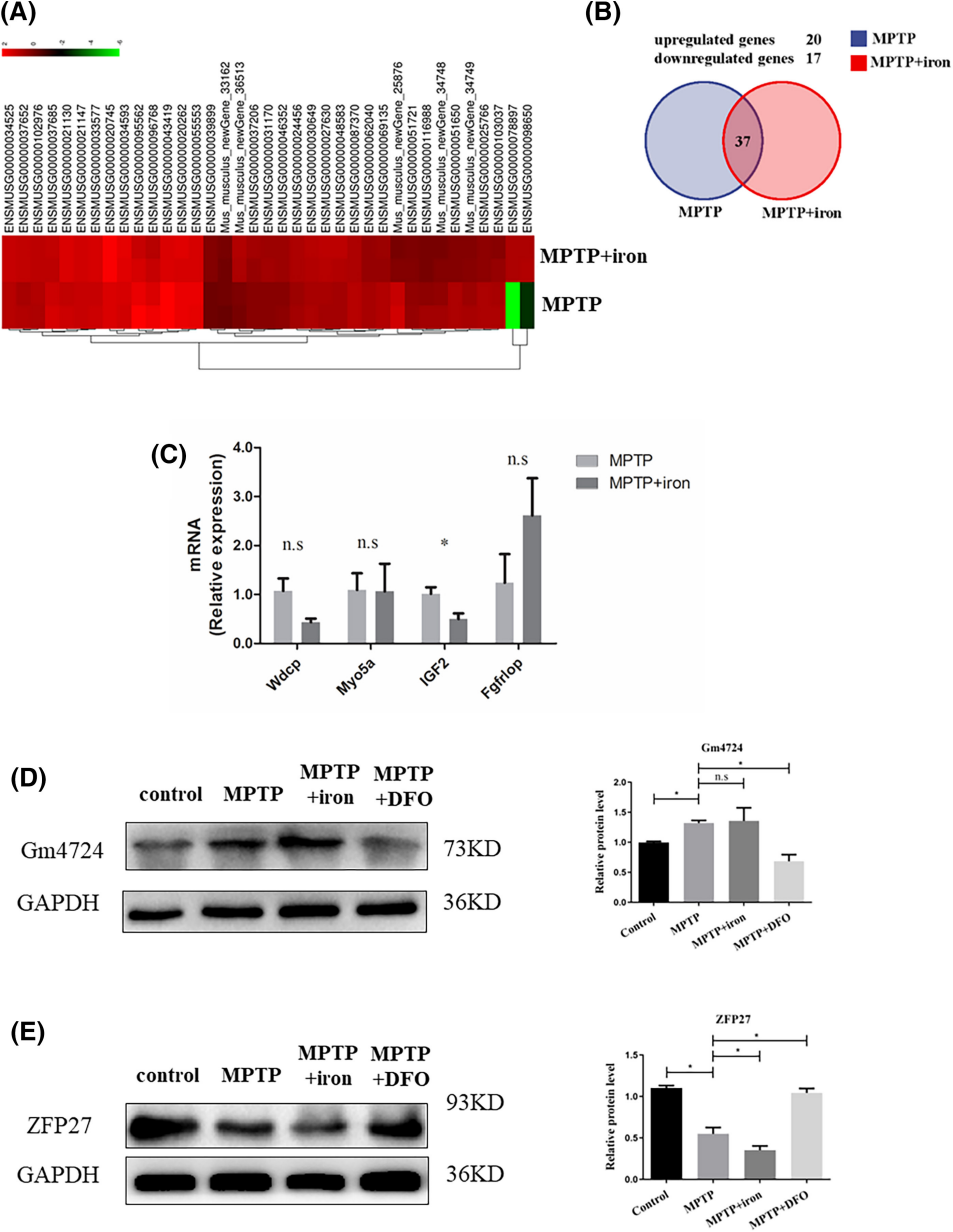
ZFP27 and IGF2 may mediate iron accumulation exacerbating PD. (A) Heatmaps display the 37 differentially expressed genes (DEGs) in the PD model mouse and iron‐treated PD mice using RNA‐seq. (B) Venn diagram for DEGs including upregulated and downregulated genes by transcriptome analysis in mice. (C) QPCR determined four kinase‐related genes in the two groups. (D) Transcription factor Gm4724 in the SN showed no significant difference in the MPTP+iron group and MPTP group (western blot). The relative grey value of Gm4724 was calculated using Image J software. (E) Transcription factor ZFP27 in the SN was less in the MPTP+iron group compared to the MPTP group (western blot). The relative grey value of ZFP27 was calculated using Image J software. All data are presented as the mean ± SEM, ns *p* ≥ 0.05; *p < 0.05.

**FIGURE 4:**
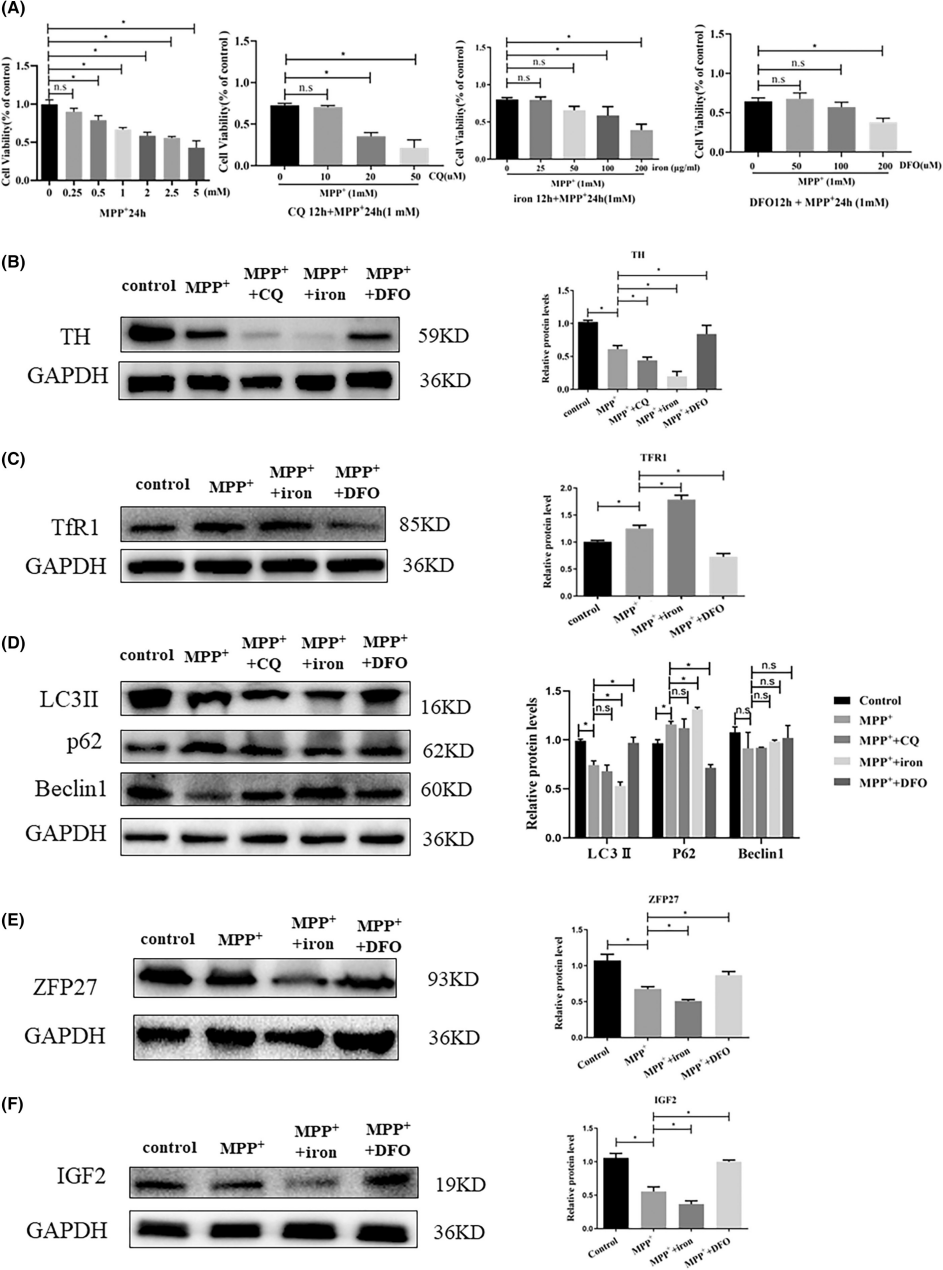
ZFP27 and IGF2 were obviously reduced with iron‐treated PD cell models. (A) The effects of various concentrations of MPP^+^, MPP^+^+iron (FAC), MPP^+^+DFO and MPP^+^+CQ on cell viability were determined using a CCK‐8 assay in SHSY5Y cells. (B–F) Western blot measuring TH, TfR1, LC3, p62, Beclin1, ZFP27 and IGF2 levels in SHSY5Y cells after MPP^+^, MPP^+^+CQ, MPP^+^+iron (FAC) or MPP^+^+DFO treatment. The relative quantification data of TH, TfR1, LC3, p62, Beclin1, ZFP27 and IGF2 relative to the internal control GAPDH were calculated using Image J software. All data are presented as the mean ± SEM. ns *p* ≥ 0.05; *p < 0.05.

To investigate how iron regulates autophagy and accelerates PD progression, 37 differentially expressed genes (DEGs) including 20 upregulated genes and 17 downregulated genes in SN were identified in PD mice and iron‐treated PD mice in the heat map and Venn by RNA‐sequence analysis (Figure 3A,B). Two transcription factors (Gm4724, ZFP27) and four kinase‐related genes (Wdcp, Myo5a, IGF2, Fgfrlop) were among these 37 DEGs. Four kinase‐related genes were increased or decreased by QPCR in the SN (Figure 3C). Next, we screened two transcription factors Gm4724, ZFP27 and kinase‐related genes IGF2. The transcription factors ZFP27 but not Gm4724 were significantly decreased in the SN of iron‐treated PD mice, and ZFP27 expression with DFO treated after PD mice showed elevated levels compared to the MPTP model (Figure 3D,E). The IGF2 antibody showed no signal in the SN. Hence, to ascertain which gene mediates iron‐inhibited autophagy in PD, we established a PD cell model in SH‐SY5Y cells. As shown in Figure 4A, SH‐SY5Y cell lines were treated with various concentrations of iron (ferric ammonium citrate, FAC), DFO and CQ for 12 h (Figure 4A). We observed no difference in cell viability of SH‐SY5Y cells treated with 10 μM CQ, 50 μg/mL FAC and 100 μM DFO compared to the PD cell model group treated by MPP^+^ (Figure 4A). Therefore, we used 10 μM CQ, 50 μg/mL FAC and 100 μM DFO for the subsequent assays. Further, we observed that TH protein levels in the PD cell model with CQ were obviously lower than in the PD group, and markedly lower in the PD group with iron. As the reducing iron by DFO treatment, TH protein levels showed a partial recovery (Figure 4B). In the PD cell model, protein levels of TfR1 in the MPP^+^ group were remarkably higher than in the control and lower in the DFO treatment group (Figure 4C). The protein expression of LC3II levels in the iron‐treated group was decreased. The DFO treatment with PD model rescued LC3II levels of the MPP^+^ group (Figure 4D). Meanwhile, LC3II expression in MPP^+^ with the CQ group has no change compared with the PD model group (Figure 4D). ZFP27/ZNF585 in the PD cell model showed a lower trend than in the control, the expression of DFO‐treated cells was higher compared to the PD model with no treatment (Figure 4E). IGF2, a member of the insulin family of polypeptide growth factors was obviously restrained compared to the control group. A decrease in protein levels in the iron‐treated PD group was observed relative to that of the PD model groups, and treatment with DFO increased the expression of IGF2 (Figure 4F). These data of mouse and cell PD model suggest that iron functions via transcription factor ZFP27 and kinase activator IGF2 to inhibit autophagy during the progression of the PD model.

### 3.4 Iron negatively regulates ZFP27 to reduce autophagy flux

**FIGURE 5:**
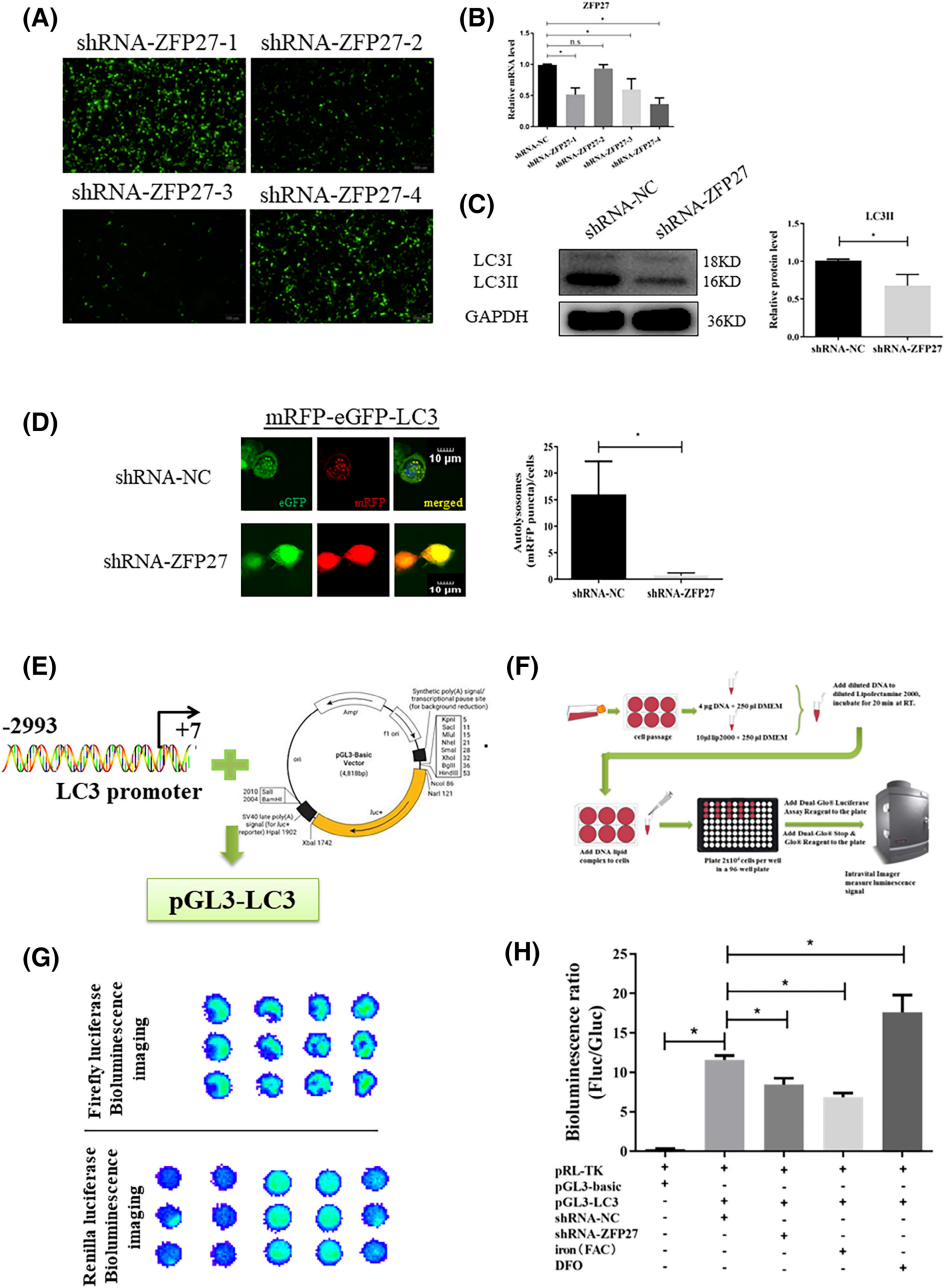
Iron‐inhibited autophagy by ZFP27 reduction. (A) The four shRNA plasmids showed different transfection efficiency in HEK293T cell lines. (B) The relative mRNA levels of ZFP27 were analysed. (C) Knockdown of ZFP27 markedly decreased the LC3II protein levels by western blot, and the relative grey intensity of LC3II relative to GAPDH was analysed using Image J. (D) Knockdown of ZFP27 had a large reduction of autophagic vacuole (green punctas) and lysosomes (red punctas) (scale bar: 10 μm), and the autolysosomes (mRFP puncta) cells were analysed using Image J. (E) LC3 promoter (−2993/+7) was fused to the pGL3‐basic vector for generating a reporter fusion (pGL3‐LC3). (F) The flow chart of the luciferase reporter assay for the promoter activity of LC3 using an in vivo bioluminescence imaging system. (G) Comparison of firefly and renilla luciferase bioluminescence imaging of empty vectors, pGL3‐LC3, ZFP27 shRNA, iron (FAC) and DFO treatments by vivo bioluminescence imaging system. (H) The luciferase intensity relative to renilla luciferase intensity showed that inhibition of ZFP27 expression reduced LC3 promoter activity; the bioluminescence ratio (Fluc/Gluc) was analysed using Image J. Triplicate plates were used to calculate the mean fold induction of transcriptional activity. All data are presented as the mean ± SEM, ns *p* ≥ 0.05; *p < 0.05.

**FIGURE 6:**
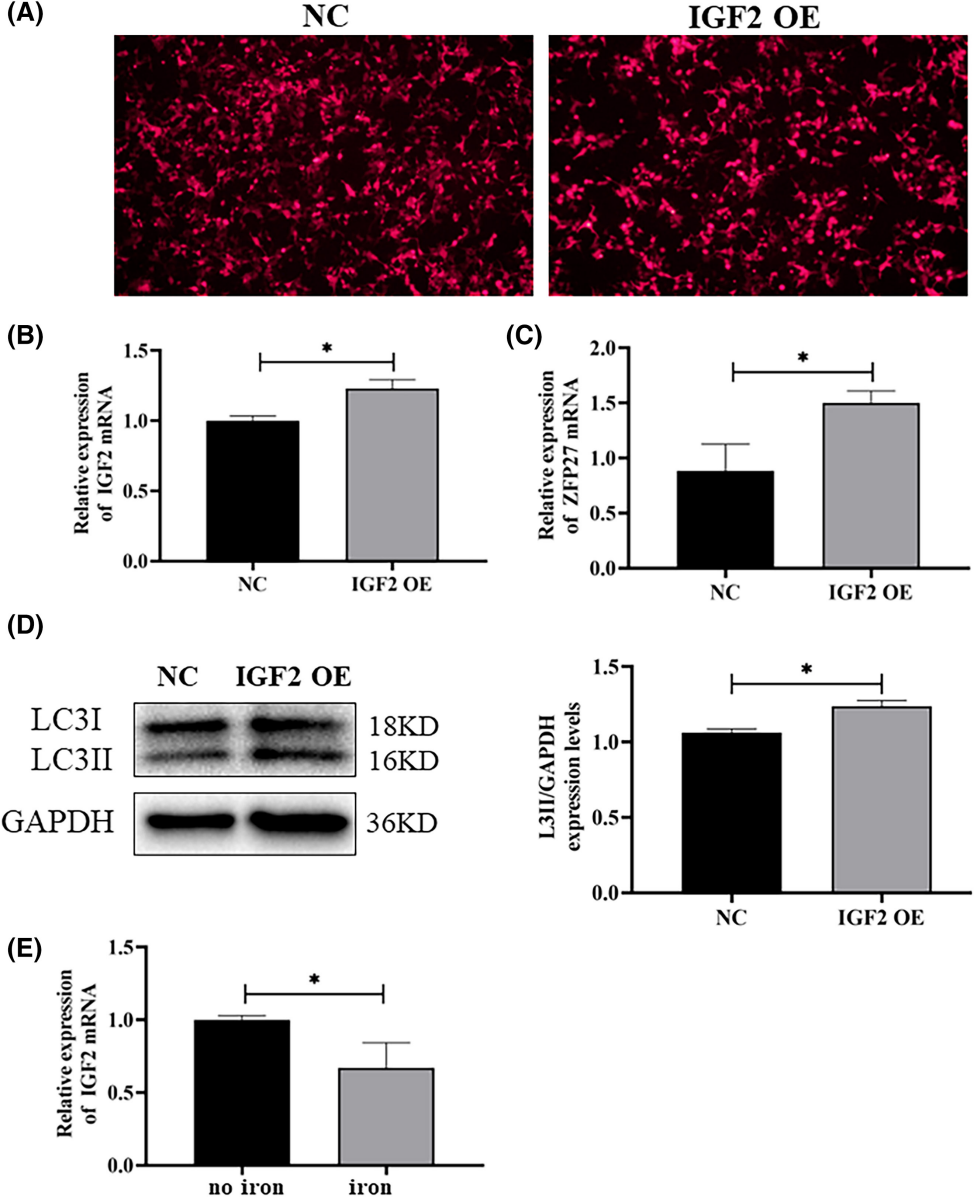
IGF2 overexpression increased ZFP27 to activate autophagy, and IGF2 expression was inhibited by iron. (A) Negative control (NC) and IGF2 overexpression (IGF2 OE) by pLVX‐IRES‐mCherry showed the transfection efficiency in HEK293T cell lines. (B, C) After plasmid transfection in HEK293T cells, IGF2 OE significantly increased the mRNA levels of IGF2 and ZFP27 compared to NC. (D) IGF2 OE markedly increased the LC3II protein levels by western blot, and the band intensity of LC3II relative to GAPDH was analysed using Image J. (E) IGF2 mRNA levels in IGF2 overexpressed cells were restrained with iron (FAC). All data are presented as the mean ± SEM, ns *p* ≥ 0.05; *p < 0.05.

**FIGURE 7:**
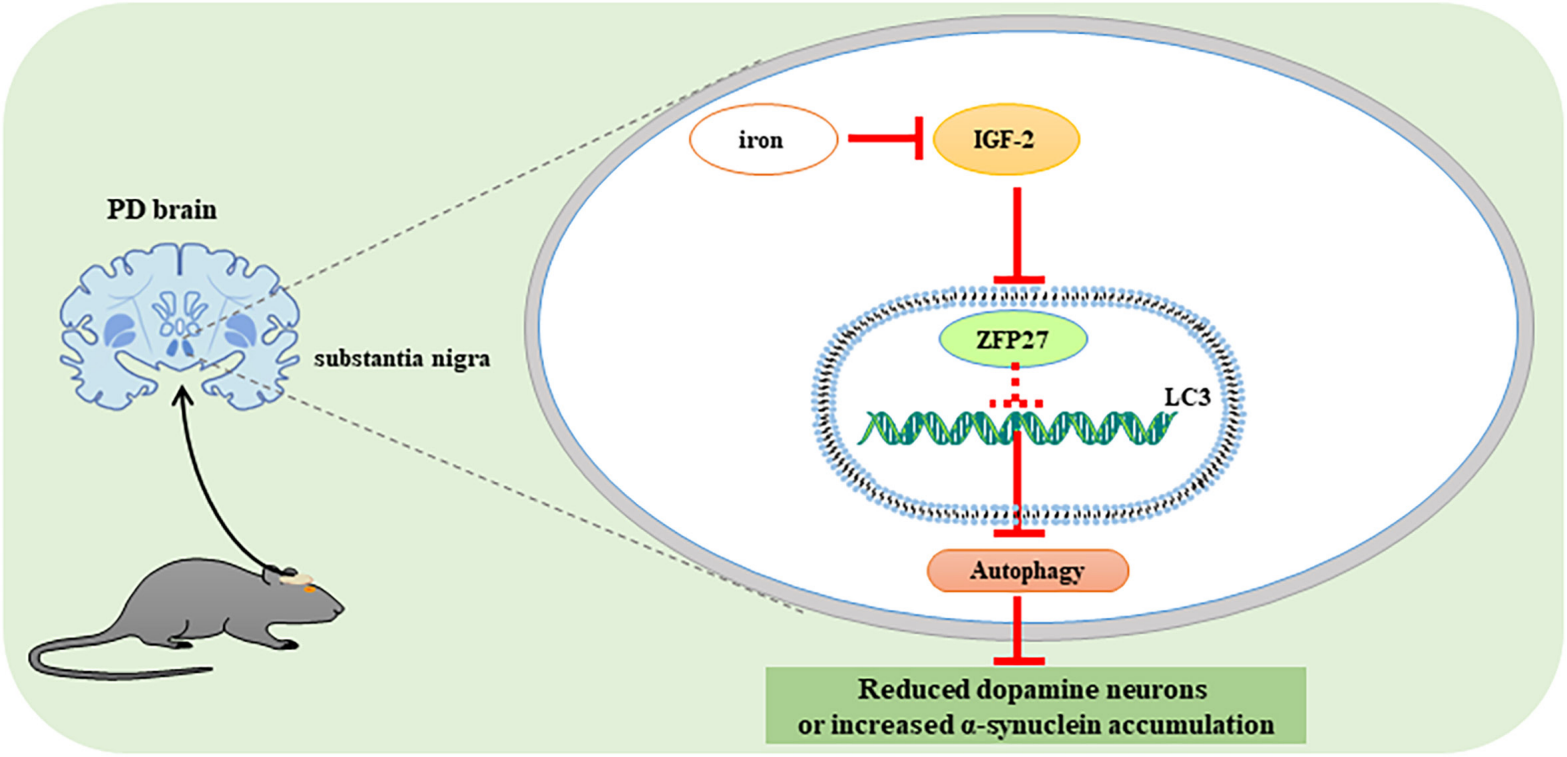
A proposed model for iron inhibition of autophagy in PD mice. In PD mice, accumulated iron suppressed kinase activator IGF2 and transcription factor ZFP27 to reduce LC3‐induced autophagy, finally increased the death of dopamine neurons and α‐syn accumulation in substantia nigra.

To gain further insight into the relationship between the transcription factor ZFP27/ZNF585 and autophagy, we first designed four shRNAs targeting ZFP27/ZNF585 to screen which shRNA‐ZFP27 could obviously lessen the expression of ZFP27 in HEK293T cells (Figure 5A,B). The data showed that shRNA‐ZFP27‐4 had higher transfection and knockdown efficiency. Then, it was determined that knock down ZFP27 via shRNA‐ZFP27‐4 could markedly decreased the LC3II protein levels in the PD cell model (Figure 5C). To further confirm whether ZFP27 regulates autophagy flux, the mRFP‐eGFP‐LC3 reporter plasmid was transfected into HEK293T cells to detect autophagy flux.^45^ We found that knocking down ZFP27 resulted in a large reduction of autophagic vacuole (green punctas) and lysosomes (red punctas) (Figure 5D). To affirm that the promoters that regulate the transcription factor ZFP27 to initiate autophagy in HEK293T cells, we tested the relationship of ZFP27 and the autophagy gene promoter using a double luciferase reporter gene assay (Figure 5E,F). The change of autophagy genes in the cell model showed that LC3II and p62 were consistent with levels in the mouse. We obtained the LC3 promoter (−2993/+7) to fuse to the pGL3 basic promoter vector for generating a reporter fusion (pGL3‐LC3) containing the LC3 with the pGL3‐Basic vector (Figure 5E). Then, we performed the luciferase reporter assay for the promoter activity of LC3 using an in vivo bioluminescence imaging system (Figure 5F). As shown in Figure 5G,H, we observed a dramatic rise after transfecting the construct pGL3‐LC3 into HEK293T cells, while knockdown of ZFP27 expression with shRNA‐ZFP27‐4 in HEK293T cells significantly reduced luciferase activities of the LC3 promoter (Figure 5G,H). The luciferase activities of iron‐treated cells were much lower than in pGL3‐LC3 with shRNA‐NC group. ZFP27 overexpression by DFO treatment indeed triggered the promoter activities of the LC3 gene (Figure 5G,H). Furthermore, to verify the role of IGF2, IGF2 was cloned and inserted into the lentiviral vector pLVX‐IRES‐mCherry. After transfected with the negative control (NC) and pLVX‐IRES‐mCherry‐IGF2, the IGF2 mRNA level in IGF2 overexpression (IGF2 OE) cells was significantly increased compared to the NC cells (Figure 6A,B). And, with IGF2 overexpression, we also observed that the ZFP27 mRNA level was higher than that of NC (Figure 6C). In addition, the LC3II protein levels in the IGF2 OE group were significantly raised relative to NC group (Figure 6D). Meanwhile, we treated the IGF2 OE cells with or without iron (FAC), and found that iron could obviously inhibit IGF2 expression (Figure 6E). Taken together, these results suggest that ZFP27 activate the LC3 promoter to enhance autophagy flux, and iron restrains IGF2 to reduce ZFP27 in PD (Figure 7).

Here, iron overload suppressed autophagy by reducing the transcription factor ZFP27 to increase α‐syn accumulation and the death of dopamine neurons in the PD model. ZFP27 was inhibited via decreased kinase activator IGF2 in the PD model.

## 4 DISCUSSION

PD continues to be a world challenge for ageing population due to its complicated pathogenesis. In PD, brain iron accumulation has long been recognized as a common feature. Nonetheless, no current therapy is based on the mechanisms of iron accumulation. Autophagy maintains neuronal homeostasis by degrading cytosolic components including misfolded proteins and dysfunctional organelles in neurodegenerative diseases.^21^ Conversely, lack of autophagy leads to autophagosome formation failure, promotes aggregation of intracellular components and becomes a source of toxic products.^21^ Neural precursor cell‐specific knockout ATG5 mice revealed loss of Purkinje cells and cerebral cortical pyramidal cells, axonal swelling and accumulations of ubiquitin‐positive inclusion bodies in numerous brain regions including the cerebral cortex, hippocampus, striatum and the nucleus gracilis.^46^ Downregulation of Atg5 or Beclin1 in human neuroblastoma cells M17 lead to α‐syn aggregations.^47^ Moreover, inactivation of autophagy in mice harbouring dopamine neuron‐specific deletion of Atg7 caused dopaminergic neuron loss, p62 accumulation, and Lewy body formation.^48^, ^49^ However, activated autophagy may be detrimental when numerous undergraded autophagic vacuoles accumulate in neurodegenerative diseases.^21^ Currently, autophagy regulation via autophagic components is challenging. The optimal approach should match different activating pathways when autophagy fails in neurodegeneration. Our study demonstrated that iron deposition inhibited kinase activator IGF2 and transcription factor ZFP27 to reduce autophagy in PD. ZFP27 is orthologous to the human gene ZNF585 and is ubiquitously expressed in the central nervous system, and even in the whole brain.^50^ The gene ontology of ZFP27/ZNF585 functions include nucleic acid binding (GO:0003676), metal ion binding (GO:0046872), transcription and DNA‐templated (GO:0006351). Our data indicated that ZFP27/ZNF585 which suppressed the LC3 promoter activity acted as a transcription repressor after iron treatment to inhibit LC3‐induced autophagy. These findings suggest that transcription factor ZFP27/ZNF585 is a potential target to increase autophagy to reduce the death of dopaminergic neurons and aggregate α‐syn in PD associated with iron accumulation.

IGF2 is a kinase‐related gene, and one of its molecular functions is protein serine/threonine kinase activator activity (GO:0043539). Case in point, kinase activator IGF2 binds to IGF‐1 receptors (a receptor tyrosine kinase) to activate PI3K/Akt or mitogen‐activated protein kinase pathway to promote proliferation.^51^, ^52^ IGF2 is a neurotrophic factor that plays a crucial role in hypoxic–ischemic brain injury, ALS, autism, AD and PD. For instance, IGF2 induces Akt phosphorylation, glycogen synthase kinase‐3β phosphorylation and β‐catenin levels to protect motor neurons in ALS.^51^ IGF2 counteracts the effects of fibroblast growth factor (FGF‐2) in inducing neuronal lineage, enhancing learning and memory as an AD drug.^53^ IGF2 depends on the IGF2 receptor hampering oxidative stress and normal mitochondrial function against degeneration of dopamine neurons and behaviour deficits.^54^ Thus, IGF2 has been proposed to be a compelling biomolecule related to PD. Serum IGF2 levels were significantly lower in PD patients than in healthy humans, and IGF2 levels positively correlated with autophagy.^55^ These reports and our findings suggest that reduced IGF2 may modulate transcription factor ZFP27 to suppress autophagy in the PD mice model. Further studies will be required to elucidate the kinase activated by IGF2 and the complex interaction between IGF2 and ZFP27 at the molecular level to identify therapeutic targets for PD.

## 5 CONCLUSIONS

In conclusion, we found that iron‐inhibited kinase activator IGF2 and transcription factor ZFP27 to reduce LC3II expression to suppress autophagy, and to increase the death of dopamine neurons or α‐syn deposition in the PD model. Hence, IGF2 and ZFP27 may be a potential therapeutic target in PD caused by iron overload.

## AUTHOR CONTRIBUTIONS

**Yinying Wang:** Conceptualization (lead); methodology (lead); writing – original draft (lead). **Qian Wen:** Formal analysis (supporting). **Rongsha Chen:** Methodology (supporting). **Zhichao Gan:** Formal analysis (supporting); methodology (supporting). **Xinwei Huang:** Data curation (supporting). **Pengfei Wang:** Methodology (supporting); project administration (supporting). **Xia Cao:** Data curation (supporting); formal analysis (supporting). **Ninghui Zhao:** Data curation (equal); funding acquisition (supporting); methodology (equal). **Zhongshan Yang:** Data curation (lead); formal analysis (lead); funding acquisition (supporting); supervision (lead). **Jinyuan Yan:** Funding acquisition (lead); project administration (lead); resources (lead); visualization (lead); writing – original draft (equal).

## FUNDING INFORMATION

This work was supported by the National Natural Science Foundation of China (Grant Numbers 31860274, 32260196); YNCUB (Grant Number 2017KF009); Yunnan Provincial Science and Technology Department (Grant Numbers 202101AT070251, 202005AC160058, 202101AZ070001‐012, 202201AY070001‐098 and ‐202201AS070084); Postgraduate Innovation Fund of Kunming Medical University (Grant Number 2022S265).

## CONFLICT OF INTEREST STATEMENT

There is no conflict of interest in this submission. I declare on behalf of my co‐authors that the work described here is original research that has not been published previously and meets the criteria for authorship. All of the authors approved the manuscript for publication.

## Supporting information


Figure S1:

## Data Availability

The data that support the findings of this study are available from the corresponding author Jinyuan Yan or the first author Yinying Wang upon reasonable request.
