# Supplementary material for: Iron‐inhibited autophagy via transcription factor ZFP27 in Parkinson's disease
Source: J Cell Mol Med. 2023 Sep 5;27(22):3614–27. doi: 10.1111/jcmm.17946 (PMC10660624; doi:10.1111/jcmm.17946)
Supplement: Supplementary file 1 — Figure S1: [file JCMM-27-3614-s001.docx]

Iron inhibited autophagy via [transcription factor](javascript:;) ZFP27 in Parkinson’s disease

**Supplemental Figure 1**

**
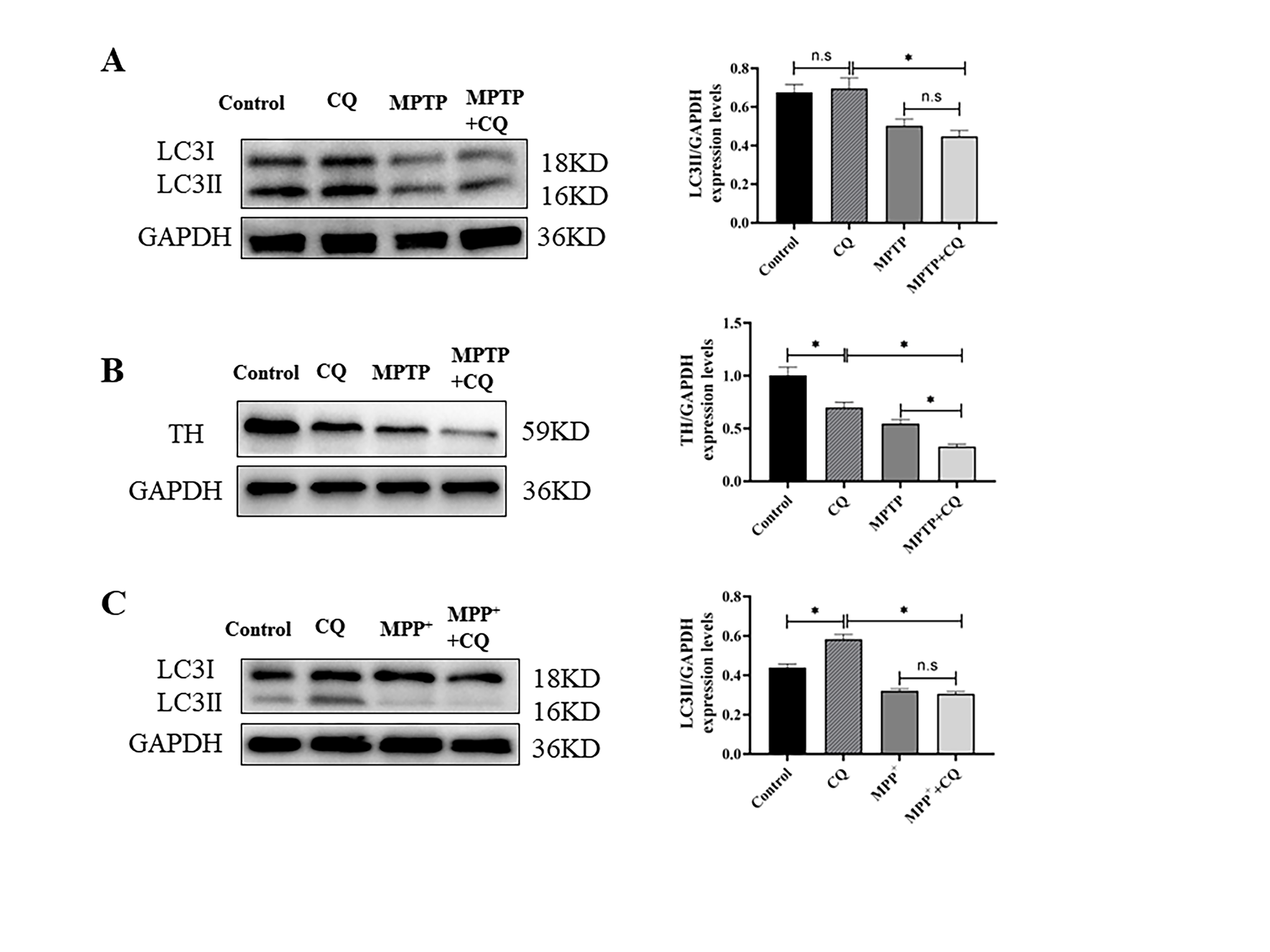
**

(A) (C) CQ administration were no significantly difference to LC3II levels in substantia nigra of control mice but increased in SH-SY5Y cell, but PD mice and cell model received CQ significantly decreased the LC3II expression by western blot ; (B) CQ treatment could remarkedly reduce TH protein level relative to the mice without CQ. And all the protein levels were quantified by Image J software. GAPDH was used as loading control in western blot assays. All data are presented as the mean±SEM, ns *P*≥0.05; **P* < 0.05.
